# *QuickStats*: Age-Adjusted Death Rates* from Lung Cancer,^^†^^ by Race/Ethnicity — National Vital Statistics System, United States, 2001–2016

**DOI:** 10.15585/mmwr.mm6730a8

**Published:** 2018-08-03

**Figure:**
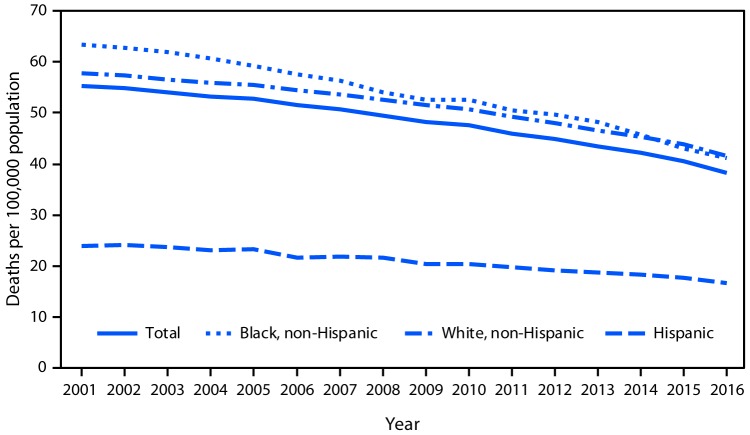
During 2001–2016, the lung cancer death rates for the total population declined from 55.3 to 38.3 as well as for each racial/ethnic group shown. During 2001–2016, the death rate for the non-Hispanic black population decreased from 63.3 to 41.2, for the non-Hispanic white population from 57.7 to 41.5, and for the Hispanic population from 23.9 to 16.6. Throughout this period, the Hispanic population had the lowest death rate.

**Sources:** CDC/National Center for Health Statistics, National Vital Statistics System, 2001–2016, Mortality. CDC Wonder online database. https://wonder.cdc.gov/ucd-icd10.html.

